# Safety and Efficacy of Aumolertinib as First‐Line Treatment in EGFR‐Mutant Lung Adenosquamous Carcinoma: A Multicenter, Single‐Arm, Prospective Phase II Study (ARISE Study)

**DOI:** 10.1002/mco2.70855

**Published:** 2026-07-22

**Authors:** Longfeng Zhang, Long Huang, Dingzhi Huang, Zhe Liu, Yongfeng Yu, Kang Miao, Qian Miao, Xiaobin Zheng, Yiquan Xu, Qian Chu, Gen Lin

**Affiliations:** ^1^ Department of Thoracic Oncology Clinical Oncology School of Fujian Medical University Fujian Cancer Hospital Fuzhou Fujian China; ^2^ Department of Lung Cancer Center The Second Affiliated Hospital of Nanchang University Nanchang Jiangxi China; ^3^ Department of Thoracic Medical Oncology Lung Cancer Diagnosis and Treatment Centre Key Laboratory of Cancer Prevention and Therapy Department of Thoracic Oncology Tianjin Medical University Cancer Institute and Hospital Tianjin China; ^4^ Department of Medical Oncology Beijing Tuberculosis and Thoracic Tumor Research Institute Beijing Chest Hospital Capital Medical University Beijing China; ^5^ Department of Medical Oncology Shanghai Chest Hospital Shanghai Jiao Tong University School of Medicine Shanghai China; ^6^ Department of Oncology Tongji Hospital Tongji Medical College Huazhong University of Science and Technology Wuhan China

**Keywords:** aumolertinib, EGFR‐mutant, first‐line treatment, lung adenosquamous carcinoma

## Abstract

Lung adenosquamous carcinoma (ASC) is a rare histological subtype of non‐small cell lung cancer (NSCLC), and prospective data regarding the efficacy of epidermal growth factor receptor (EGFR) tyrosine kinase inhibitors (TKIs) in this patient population remain scarce. This prospective, multicenter, single‐arm phase II trial (NCT04354961) evaluated the safety and efficacy of aumolertinib as first‐line treatment for treatment‐naïve patients with stage IV EGFR‐mutant ASC. Eligible patients received aumolertinib 110 mg orally once daily, with progression‐free survival (PFS) as the primary endpoint and objective response rate (ORR), disease control rate (DCR), overall survival (OS), and safety as secondary endpoints. The study was terminated early due to slow accrual, with 12 patients enrolled (median age: 66 years; 58.3% male; 58.3% with EGFR exon 19 deletion). After a median follow‐up of 29.0 months, the median PFS and OS were 11.1 months (95% CI, 4.27–NA) and 16.7 months (95% CI, 11.3–NA), respectively. Confirmed ORR and DCR were 58.3% and 83.3%. Treatment‐related adverse events (TRAEs) occurred in 75% of patients, with 33.3% experiencing grade 3–4 TRAEs; no treatment‐related deaths were observed. Aumolertinib exhibits clinical activity with a manageable safety profile in EGFR‐mutant ASC patients. However, its reduced efficacy compared with EGFR‐mutant adenocarcinoma highlights the need for histology‐specific therapeutic strategies.

**Trial Registration**: NCT04354961

## Graphical abstract

Aumolertinib treatment resulted in improved clinical activity in EGFR‐mutant ASC.

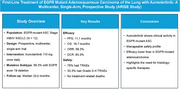

## 1 Introduction

Lung adenosquamous carcinoma (ASC), a rare and aggressive subtype of non‐small cell lung cancer (NSCLC), constitutes 0.4%–4% of all NSCLC cases [1]. ASCs are characterized by the coexistence of adenocarcinoma (AC) and squamous cell carcinoma (SqCC) components (with each accounting for ≥ 10% of the tumor volume) and have a significantly worse prognosis than pure histological subtypes [2, 3, 4]. Compared with pure AC or SqCC, resected stage I‐III ASC patients have a 5‐year survival rate of only 15%–28%, significantly lower than that of pure AC (40%–50%) or SqCC (25%–35%) [5]. For advanced‐stage ASC, platinum‐based chemotherapy remains the traditional first‐line option, but its efficacy is limited with a median overall survival (mOS) of merely 8–12 months, a median progression‐free survival (mPFS) of only 4.5–5.2 months, and substantial toxicity, leaving an urgent unmet need for more effective and tolerable therapies [6].

Early pathogenetic models suggested a multiclonal origin for ASC, suggesting independent development of AC and SqCC components [4, 7]. Morphological studies by Hammond et al. revealed spatial segregation of these components during early tumorigenesis, with progressive intermingling as the disease advances [8]. However, emerging molecular evidence has revised this view: genomic analysis by Lin et al. found identical epidermal growth factor receptor (EGFR)/KRAS driver mutations in both components in 68% of ASC cases, strongly supporting a monoclonal origin with subsequent divergent differentiation [4]. This molecular homogeneity, particularly the high prevalence of EGFR mutations (30%–51.8% in Asian populations) [9], provides a rationale for targeted therapy. Notably, EGFR mutations in ASC are similar in frequency to those in AC but much higher than in pure SqCC (< 5%) [9], with 80%–90% being common sensitive mutations (exon 19 deletion [Ex19Del] and exon 21 L858R point mutation) [10], which are known to respond to EGFR tyrosine kinase inhibitors (TKIs).

Despite the high EGFR mutation rate, ASC presents unique therapeutic challenges due to its bidirectional differentiation. While AC components are sensitive to EGFR‐TKIs, SqCC components exhibit intrinsic resistance mediated by distinct gene expression profiles (e.g., *TP53* amplification and *SOX2* overexpression) and signaling pathways [11]. Retrospective studies of first/second‐generation EGFR‐TKIs (erlotinib, gefitinib, afatinib) in EGFR‐mutant ASC have shown limited efficacy, with objective response rates (ORR) of 26.5%–56.6% and mPFS of 4.3–15 months [4, 5, 6]. Moreover, acquired resistance develops in most patients within 9–14 months, with approximately 50% harboring the T790M mutation [16], which is refractory to first/second‐generation TKIs. These limitations highlight the need for more potent EGFR‐targeted agents to address both intrinsic and acquired resistance in ASC.

Third‐generation EGFR‐TKIs (osimertinib and aumolertinib) have revolutionized the treatment of EGFR‐mutant NSCLC by irreversibly inhibiting EGFR sensitive mutations and T790M resistance mutations, while sparing wild‐type EGFR to reduce off‐target toxicity [12, 13, 14, 15, 16, 17]. Compared with osimertinib, aumolertinib, a structurally optimized third‐generation TKI approved in China for first‐line treatment of EGFR‐mutant NSCLC, exhibits comparable efficacy (ORR 73.8%, median PFS 19.3 months) [17, 18, 19, 20], with a lower incidence of treatment‐related interstitial lung disease(ILD) (0.9% vs. 4%) [17], making it a promising candidate for ASC patients who may tolerate less toxicity. Additionally, third‐generation TKIs have improved central nervous system (CNS) penetration [18], a critical advantage given that 25%–30% of advanced ASC patients develop CNS metastases [31], which is associated with poor outcomes.

However, prospective data on third‐generation EGFR‐TKIs in EGFR‐mutant ASC remains scarce. Most clinical trials of EGFR‐TKIs have focused on unselected EGFR‐mutant NSCLC populations, with ASC patients underrepresented or excluded, leading to a lack of evidence‐based treatment guidelines for this rare subtype. While retrospective studies and case reports have demonstrated promising activity of third‐generation EGFR‐TKIs in ASC, the level of evidence remains low and insufficient to guide clinical practice. The ARISE trial (NCT04354961) was therefore designed as a prospective, multicenter, single‐arm phase II study to evaluate the safety and efficacy of aumolertinib as first‐line treatment for treatment‐naïve EGFR‐mutant advanced ASC. This study aims to fill the gap in prospective evidence, validate the therapeutic value of aumolertinib in this specific population, and provide a basis for histology‐specific treatment strategies.

## 2 Results

### 2.1 Patient Characteristics

**FIGURE 1:**
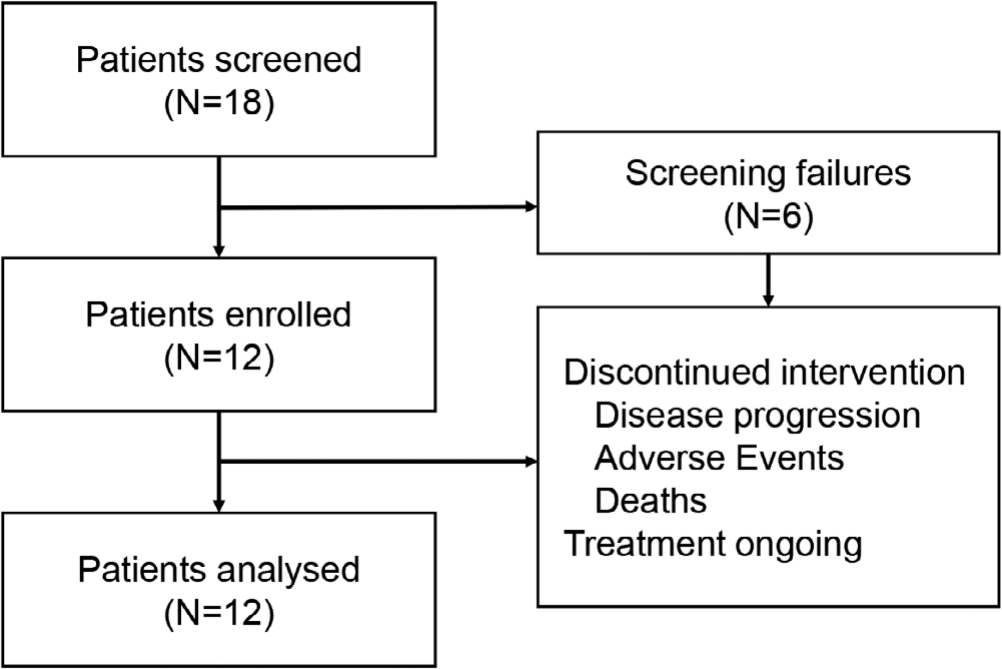
Patient disposition of the aumolertinib as first‐line treatment for EGFR‐Mutant Lung Adenosquamous Carcinoma Study (ARISE).

A total of 18 patients were recruited from six Chinese medical centers, of which 12 met the inclusion criteria. The remaining six were excluded because of consent withdrawal. The study design is depicted in Figure 1. The median age at diagnosis was 66 years (interquartile range [IQR], 57.5–68.25), with most patients being male (58.3%), and 58.3% of participants harbored EGFR exon 19 deletions. All participants had measurable diseases at baseline as per RECIST 1.1 and received at least one dose of aumolertinib. The baseline demographics are presented in Table 1. All patients had an ECOG PS score of 0 to 1, with 75% being non‐smokers; 66.7% and 33.3% were in stages IV‐B and IV‐A, respectively. Seven patients had Ex19del mutations, and the others had L858R mutations.

**TABLE 1:** Baseline demographics and patient characteristics.

Parameters	All patients (*n* = 12)
Age (years), median (range)	66 (51–71)
Gender	
Female	5 (41.7%)
Male	7 (58.3%)
ECOG PS	
0–1	12 (100%)
≥ 2	0 (0)
Smoking status	
Never smoked	9 (75%)
Former/current smoker	3 (25%)
Type of EGFR mutation	
Exon 19 deletion	7 (58.3%)
L858R	5 (41.7)
Tumor stage	
IVA	4 (33.3%)
IVB	8 (66.7%)
CNS metastases	
No	9 (75%)
Yes	3 (25%)

Abbreviations: ASC, lung adenosquamous carcinoma; CNS, central nervous system; ECOG PS, eastern cooperative oncology group performance score; EGFR, epidermal growth factor receptor.

### 2.2 Clinical Efficacy

**FIGURE 2:**
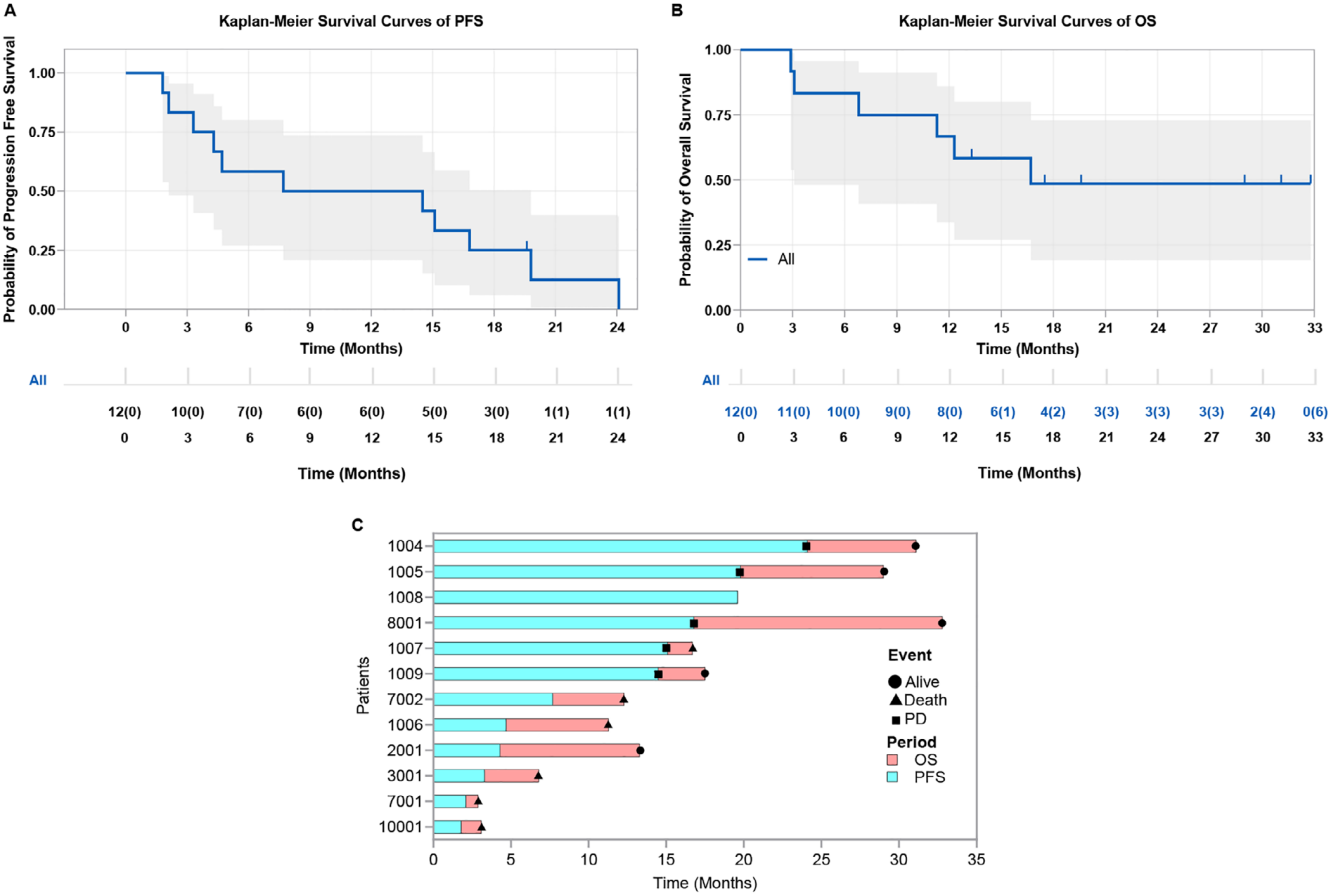
Patient survival information. (A) Kaplan–Meier curve of progression‐free survival, (B) overall survival, and (C) detailed follow‐up and survival information for all 12 patients.

**FIGURE 3:**
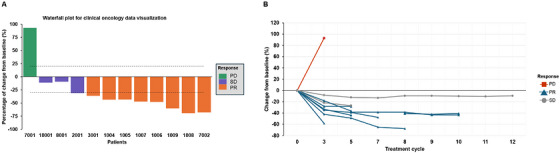
Variation of tumor size. (A) Optimal variation and (B) variation over time.

**FIGURE 4:**
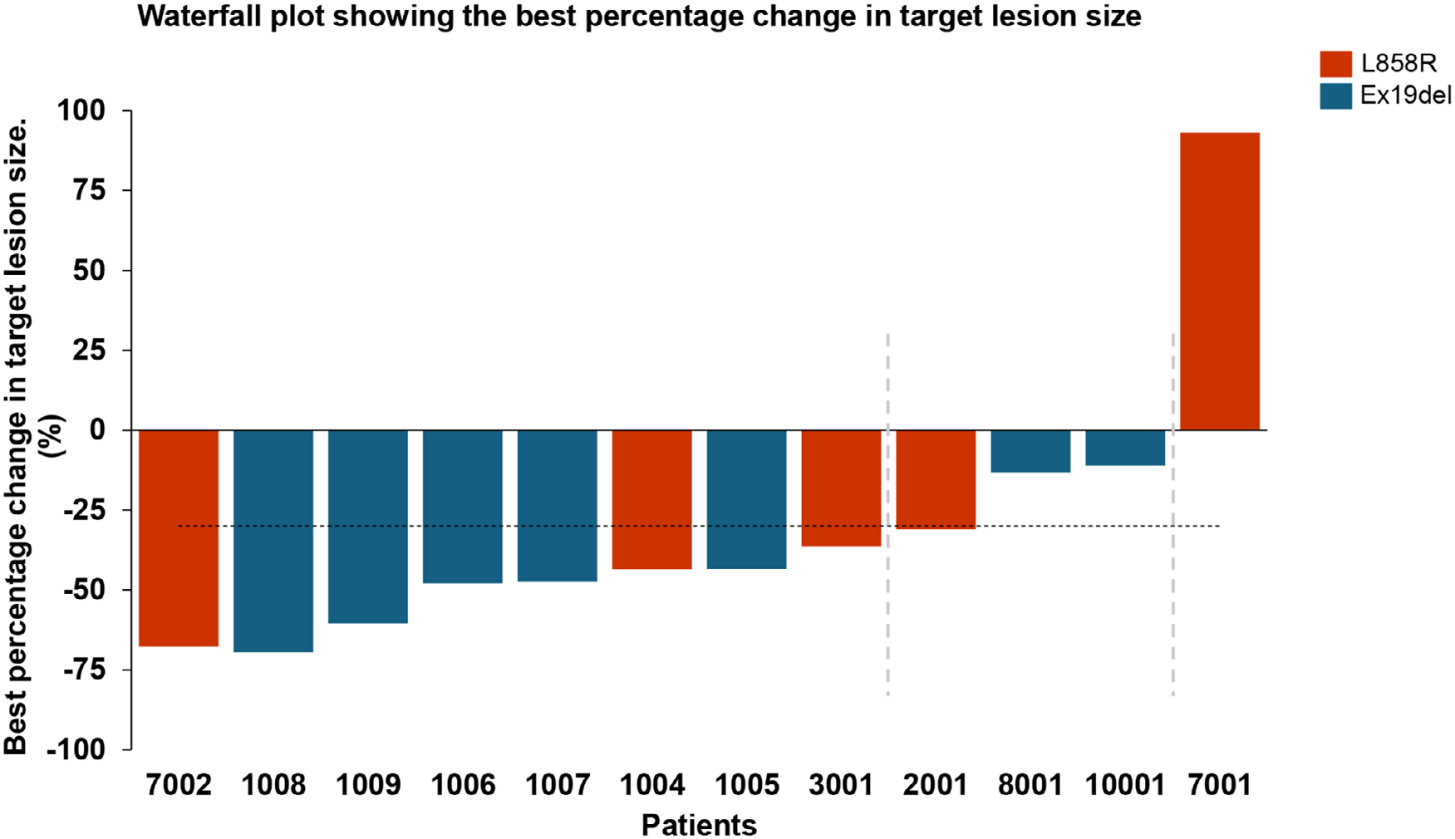
Waterfall plot showing the best percentage change in target lesion size for all evaluable patients (*n* = 12). (Each bar represents an individual patient, colored by EGFR mutation type [blue: Ex19del; red: L858R]. The dashed line indicates the threshold for partial response [PR, ≥ 30% reduction in tumor size]. Patients achieving PR [*n* = 8] are shown to the left of the dashed line, stable disease [SD, *n* = 3] in the middle, and progressive disease [PD, *n* = 1] to the right.)

As of July 2024, the median follow‐up was 29.0 months, with patients receiving 4–26 aumolertinib doses. The median PFS was 11.1 months (95% CI, 4.27–NA; Figure 2A). Notably, PFS rates at 3 and 6 months reached 70.3% (95% CI, 52.8–82.3) and 56.1% (95% CI, 38.6–70.4), respectively. At the time of analysis, the median OS was 16.7 months, with 6‐ and 12‐month survival rates of 94.2% (95% CI, 90.4–96.5) and 84.5% (95% CI, 79.3–88.5), respectively (Figure 2B). Efficacy assessment of all 12 patients revealed that eight (66.67%) achieved PR, three (25.0%) had SD, and one (8.33%) experienced PD, with none of them having achieved CR (Figures 2C, 3 and 4). The confirmed ORR was 58.3% (95% CI, 62.6–74.6), and the DCR was 83.3% (95% CI, 89.6–96.2). Importantly, among three patients with baseline CNS metastases, one achieved intracranial CR, highlighting the efficacy of aumolertinib.

To further explore the efficacy of aumolertinib in different EGFR mutation subtypes, we performed a post hoc subgroup analysis based on mutation type (Ex19del vs. L858R). Among seven patients with Ex19del mutation, the median PFS was 15.10 months (95% CI, 4.70–NA) and median OS was NA (95% CI, 11.30–NA), with a confirmed ORR of 71.4% (5/7) and DCR of 85.7% (6/7). For five patients with L858R mutation, the median PFS was 4.27 months (95% CI, 3.33–NA), median OS was 12.30 months (95% CI, 6.80–NA), with a confirmed ORR of 40.0% (2/5) and DCR of 80.0% (4/5). Although the small sample size limits statistical inference, Ex19del mutation was associated with numerically better efficacy outcomes compared with L858R mutation, consistent with prior findings in EGFR‐mutant adenocarcinoma (Table S1)

### 2.3 Safety

Among the 12 patients, 75% (9/12) experienced at least one treatment‐related adverse event (TRAE). The most frequent TRAEs was elevated AST (25.0%). Grade 3–4 TRAEs occurred in three patients (25.0%), including neutropenia, hyponatremia, and hypokalemia. Other AEs included irregular vaginal bleeding, hyperuricemia, azotemia, hypercholesterolemia, myalgia, thrombocytopenia, leukopenia, hyperbilirubinemia, hypokalemia, hypoalbuminemia, hypocalcemia, and elevated blood creatine phosphokinase. No treatment‐related deaths were observed (Table 2; Figure S1).

**TABLE 2:** Summary of AEs and TRAEs.

Adverse events	N (%)
All AEs	9 (75)
Grades 1–2	9 (75)
Elevated ALT	2 (16.7)
Elevated AST	3 (25.0)
Pain	2 (16.7)
Anemia	1 (8.33)
Others	8 (66.7)
Grade 3	3 (25.0)
Neutropenia	1 (8.33)
Elevated GGT	1 (8.33)
Hypokalemia	1 (8.33)
Grade 4	1 (8.33)
Hyponatremia	1 (8.33)

TRAEs	All AEs	Grades 3–4 AEs
Total	9	4
Irregular vaginal bleeding	1	0
Neutropenia	1	1
Hyperuricemia	1	0
Azotemia	1	0
Myalgia	3	0
AST/ALT elevation	3	0
Thrombocytopenia	1	0
Anemia	1	0
Hyperbilirubinemia	3	0
Hypercholesterolemia	1	0
Hypoalbuminemia	1	0
Hypocalcemia	1	0
Elevated GGT	0	1
Hypokalemia	0	1
Hyponatremia	0	1
Elevated creatine phosphokinases	3	0

Abbreviations: AE, adverse event; ALT, alanine aminotransferase; AST, aspartate aminotransferase; FAS, full analysis set; GGT, gamma‐glutamyl transferase; TRAE, treatment‐related adverse event.

## 3 Discussion

This study was initially designed as a randomized controlled study (RCT). However, due to the historically suboptimal efficacy of chemotherapy in ASC, most eligible patients declined participation. Consequently, the study was adapted into a single‐arm phase II trial evaluating the clinical efficacy of aumolertinib in treatment‐naïve EGFR‐mutant ASC. Our findings demonstrated a median PFS of 11.1 months (95% CI, 4.27–NA), a 2.3‐fold improvement over historically reported chemotherapy outcomes [17], with an ORR of 58.3% and a 12‐month OS rate of 84.5%. These findings suggest that the third‐generation EGFR‐TKI may represent a new therapeutic benchmark for this rare and aggressive histological subtype.

For advanced EGFR‐wildtype ASC, platinum‐based doublet chemotherapy (e.g., cisplatin/pemetrexed and carboplatin/paclitaxel) remains the first‐line standard of care, with limited efficacy data specifically for EGFR‐mutant ASC. Retrospective studies have reported that advanced ASC patients receiving platinum‐based chemotherapy achieve a median PFS of only 4.5–5.2 months and median OS of 8–12 months, with ORR ranging from 20% to 35% [6, 42]. A multicenter real‐world study of 86 advanced ASC patients (including 18 EGFR‐mutant cases) showed that chemotherapy yielded a median PFS of 5.1 months and OS of 9.8 months, with no significant survival difference between EGFR‐mutant and wild‐type subgroups due to the intrinsic resistance of squamous components [43].

Chemo‐immunotherapy (e.g., platinum‐based chemotherapy plus PD‐L1 inhibitors) has emerged as a standard option for advanced NSCLC, but its role in ASC remains unproven due to limited data. A small retrospective cohort (*n* = 32) demonstrated that chemo‐immunotherapy achieved a median PFS of 6.3 months and OS of 13.2 months in advanced ASC, with ORR of 37.5% an improvement over chemotherapy but still inferior to our study's outcomes [44]. Notably, EGFR‐mutant NSCLC patients are generally less responsive to immunotherapy due to low tumor mutational burden and immunosuppressive tumor microenvironment [45], making chemo‐immunotherapy a suboptimal option for this subgroup.

In contrast, our study with aumolertinib achieved a median PFS of 11.1 months (95% CI, 4.27–NA) and median OS of 16.7 months (95% CI, 11.3–NA), representing a 2.2‐fold improvement in PFS and 1.4‐ to 2.1‐fold improvement in OS compared with platinum‐based chemotherapy, and a 1.8‐fold improvement in PFS and 1.26‐fold improvement in OS compared with chemo‐immunotherapy. The confirmed ORR of 58.3% with aumolertinib is also substantially higher than the 20%–37.5% observed with standard of care [42, 44]. These data strongly support that aumolertinib offers superior efficacy to standard chemotherapy and chemo‐immunotherapy for EGFR‐mutant advanced ASC, addressing an unmet need in this rare and aggressive subtype.

To our knowledge, this is the first prospective clinical trial to evaluate the efficacy and safety of aumolertinib in patients with EGFR‐mutant ASC and EGFR mutations, with favorable results, aligning with the expectations based on its efficacy in AC. Although third‐generation EGFR‐TKIs have shown robust efficacy in lung AC, their role in ASC has remained unclear [18, 19, 20, 21, 22, 23, 24]. Most previous studies focused on the EGFR‐mutated population, whereas the current study reports data exclusively from patients with ASC alone. Another study revealed that treatment with gefitinib and erlotinib showed an mPFS and ORR of 8.7 months and 71.4% [26]. Previous studies of first‐ and second‐generation EGFR‐TKIs in ASC reported median PFS ranging from 8.08 to 10.1 months and ORR above 30% [4, 27]. A key efficacy comparison of EGFR‐TKI therapies for ASC is summarized in Table S2. Our findings confirm that aumolertinib provides meaningful clinical benefit with favorable median PFS and no new safety signals.

This study demonstrated the beneficial effect of EGFR‐TKI treatment in ASC, albeit with slightly reduced efficacy compared with pure AC. For instance, the AENEAS trial reported a median PFS of 19.3 months in EGFR‐mutant AC, whereas our study observed 11.1 months in ASC. This aligns with prior reports indicating that ASC has worse outcomes than both AC and SqCC [15]. A cervical cancer study also found that ASC patients had significantly lower 5‐year disease‐free survival (47.8%) compared with SCC patients (67.6%) [25]. These comparisons reinforce the aggressive nature of ASC and the need for histology‐specific treatment strategies.

In lung ASC patients with brain metastases, a median time to brain metastasis (TTB) of 5.7 months and an OS of 13.8 months have been reported. Longer TTB (> 12 months), surgical resection of brain metastases, and receipt of more than three chemotherapy cycles (which extended median OS to 7.9 months) were associated with improved survival. In a separate study of ASC, EGFR mutations were identified in 21.9% of patients, including exon 19 deletion and exon 21 L858R mutation, with no significant survival difference observed between mutation subtypes [28]. Notably, from the ARISE trial, patients with EGFR 19del (58.3%) derived more favorable survival benefits than those with L858R, showing an mPFS of 15.1 months and prolonged mOS that had not yet been reached. Among three patients with baseline CNS metastases, one achieved intracranial complete response.

The numerical difference in efficacy between the exon 19 deletion and L858R subgroups (ORR 71.4% vs. 40.0%) is consistent with the known biological heterogeneity of EGFR mutations. Preclinical studies have demonstrated that exon 19 deletions lead to stronger EGFR kinase activation and greater sensitivity to EGFR‐TKIs compared with L858R mutations, which may explain the more favorable outcomes in the exon 19 deletion subgroup. However, the small sample size (*n* = 5 for L858R) limits the generalizability of this observation, and larger cohorts are required to validate this subgroup difference.

The reduced efficacy of EGFR‐TKIs in ASC relative to AC may be attributed to the biological heterogeneity of the tumor. ASC contains both glandular and squamous components, each with distinct gene expression profiles and signaling pathways [29]. Molecular studies have shown that ASC may harbor mutations common to both AC (e.g., *EGFR* and *KRAS*) and SqCC (e.g., *TP53* and *SOX2* amplification). Single‐cell RNA sequencing has revealed transcriptional plasticity in ASC, with AC cells undergoing squamous trans differentiation via *Notch1/SOX2* coactivation [29]. This transition is characterized by downregulation of AC markers (TTF1, Napsin A) and upregulation of squamous markers (p63, DSG3), creating a microenvironment less responsive to EGFR inhibition [29].

Recent studies have shown that KRAS G12C mutations, which are present in both AC and SqCC, activate the PI3K‐AKT‐mTOR and RAS‐MEK‐ERK pathways, contributing to resistance. Emerging molecular evidence supports a phenotypic plasticity continuum in which AC cells undergo squamous trans differentiation via *Notch1/SOX2* coactivation, generating hybrid clones with reduced EGFR dependence [30]. Such molecular reprogramming establishes a therapeutic gradient: SqCC < ASC < AC, consistent with our trial's PFS of 11.1 months and corroborating previous ASC‐specific data (10.1 months) [17].

These findings suggest that ASC is not merely a histological hybrid but a biologically distinct entity with unique therapeutic challenges. Histologically, ASC consists of two distinct tumor cell types—AC and SqCC—each potentially exhibiting different gene expression patterns and active signaling pathways [31]. Despite sharing EGFR mutations as a common oncogenic driver, the dual lineage composition may modify tumor responsiveness to EGFR‐TKIs compared with pure AC. Classic EGFR mutations associated with favorable outcomes, such as exon 19 deletions and L858R substitutions, may also occur at different frequencies in ASC than in pure AC [32, 33].

To improve outcomes in ASC, future strategies should explore combination therapies that target both histological components. EGFR‐TKI plus chemotherapy regimens have shown promise in overcoming resistance and improving PFS in NSCLC. Studies such as FLAURA‐2 demonstrated that combining osimertinib with chemotherapy significantly prolonged PFS compared with monotherapy. In addition, platinum‐based regimens with pemetrexed have shown synergy by targeting both AC and squamous components [34]. These approaches may be particularly beneficial in ASC, where dual lineage features complicate monotherapy efficacy.

Incorporating molecular profiling and single cell sequencing into clinical decision‐making could further refine treatment selection and identify resistance mechanisms early. Dual inhibition strategies, such as targeting KRAS G12C and mTOR, have also shown synergistic effects in preclinical models of both AC and SqCC and may hold promise for ASC.

Currently, no ASC‐specific treatment regimen is approved, and management follows NSCLC guidelines such as NCCN. For adults with metastatic NSCLC harboring EGFR exon 19 deletion or exon 21 L858R mutation, the NCCN guidelines recommend osimertinib monotherapy as well as osimertinib combined with platinum and pemetrexed as first‐line options [12]. According to the 2024 ASCO Living Guideline, osimertinib is strongly recommended as first‐line therapy for classical EGFR mutations, with the optional addition of platinum doublet chemotherapy or amivantamab plus lazertinib in selected patients [35].

Although the landmark FLAURA trial's reported a median PFS of 18.9 months for osimertinib [36], whereas the present ARISE study demonstrated an mPFS of 11.1 months and ORR 58.3% in treatment‐naïve EGFR‐mutant ASC, the FLAURA patient cohort consisted predominantly adenocarcinoma NSCLC and not ASC. Moreover, patients with advanced or metastatic NSCLC treated with chemotherapy‐based regimes had an mPFS of ∼5 months and mOS was 11.20 months. Such chemotherapy regimens were associated with considerable toxicity, frequently leading to early treatment discontinuation [37]. Notably, the present study provides the first prospective evidence supporting the use of third‐generation EGFR‐TKIs in this rare histologic subtype.

Combination regimens combining EGFR‐TKIs and chemotherapy have proven highly effective in multiple settings [38, 39, 40]. These included the AENEAS‐2 [41], EVIDENCE [42], IMPRESS [43], and the others [44, 45], which consistently demonstrate superior PFS compared with monotherapy. For instance, the FLAURA‐2 trial evaluated the combinatory effect of osimertinib with chemotherapy and showed significantly prolonged PFS compared with osimertinib alone [44]. The distinct clinical behavior of ASC underscores the need for future investigations of combination strategies to improve efficacy and overcome resistance [15].

Our findings are consistent with and extend prior retrospective data on EGFR‐TKI therapy in ASC. A multicenter retrospective study (n = 129) reported an ORR of 56.6% and mPFS of 10.1 months with first/second‐generation TKIs [7], whereas our study using third‐generation aumolertinib achieved a numerically higher ORR (58.3%) and longer mPFS (11.1 months), highlighting the potential advantage of third‐generation TKIs in this population. Compared with a small prospective study of osimertinib in ASC (*n* = 8, mPFS 9.7 months) [28], aumolertinib showed comparable efficacy and a favorable safety profile, with no grade 4 ILD observed. Table S2 summarizes key efficacy outcomes of published EGFR‐TKI trials in ASC, confirming that aumolertinib represents a promising and competitive treatment option for this rare subtype.

This study has several limitations. The single‐arm design, which was necessary due to the rarity of ASC and high rates of chemotherapy refusal, precludes direct head‐to‐head comparisons with standard treatments. The small sample size (*n* = 12) limits robust subgroup analyses, such as comparisons exon 19 deletions and L858R mutations. Nevertheless, our findings offer valuable clinical insights and established histology‐specific benchmarks for EGFR‐mutant ASC, providing a foundation for future larger cohort trials and combination therapy studies.

## 4 Conclusion

Aumolertinib shows clinically significant activity in EGFR‐mutant ASC, though its reduced efficacy compared with ACs highlights the need for histology‐tailored therapeutic options.

## 5 Methods

### 5.1 Patients

Eligible patients aged 18–80 with histologically confirmed metastatic or recurrent lung ASC harboring EGFR mutations were enrolled. EGFR mutation status was confirmed by next‐generation sequencing (NGS) or polymerase chain reaction (PCR) in tumor tissue or plasma/blood specimens. In this trial, EGFR‐mutant status was defined as mutations known to be sensitive to third‐generation EGFR‐TKIs, specifically exon 19 deletion (19Del) and exon 21 L858R substitution. EGFR amplification, T790M resistance mutations, exon 20 insertions, and other rare EGFR mutations (e.g., G719X and L861Q) were explicitly excluded.

To minimize heterogeneity and biased inferences within the small sample size of 12 patients, only tumors harboring 19Del or L858R were eligible.

Furthermore, because all enrolled patients were treatment‐naïve, those with T790M mutation were excluded, as this mutation confers acquired resistance following prior treatment with earlier‐generation EGFR‐TKIs.

As per the modified WHO classification for unresectable disease, diagnostic criteria for advanced/recurrent diseases required prior confirming ASC for recurrent cases, with biopsy specimens confirming both AC and SqCC components (each ≥ 10% of tumor volume) for treatment‐naïve cases in advanced cases. Key inclusion criteria included ECOG performance status (ECOG PS) of 0–2, with at least one measurable lesion as per RECIST 1.1 and asymptomatic CNS metastases. Additional patients with stage IIIB‐IV ASC or AC without prior systemic therapy and the completion of neoadjuvant/concurrent chemotherapy ≥ 6 months pre‐enrollment were also included. Patients with prior EGFR inhibitor exposure, hypersensitivity to aumolertinib components, cardiac dysfunction, interstitial lung disease, inadequate hematologic function, or uncontrolled comorbidities were excluded.

### 5.2 Trial Design

Initially, the study design was planned as a randomized phase II study comparing aumolertinib to chemotherapy; the ARISE trial was amended to a single‐arm phase II design in October 2021 because of slow accrual. This prospective multicenter study enrolled patients between March 23, 2021, and January 31, 2023. The trial was conducted in accordance with the Declaration of Helsinki and the International Conference on Harmonization Good Clinical Practice Guidelines (ICH‐GCP) and was approved by the ethics committee from Fujian Cancer Hospital and all participating sites. Written informed consent was obtained from all patients before enrollment. All patients were screened across six Chinese cancer centers.

### 5.3 Treatment

Patients received oral aumolertinib, 110 mg once daily, in 21‐day cycles until disease progression, unacceptable toxicity, or withdrawal was noted. Baseline tumor staging included contrast‐enhanced CT/MRI of the chest/abdomen, and radiologic assessments were performed every 6 weeks (two cycles) and evaluated by both investigators as per RECIST 1.1 [46]. Safety monitoring was carried out in accordance with Common Terminology Criteria for Adverse Events (version 4.03; CTCAE v4.03), with all treatment‐emergent adverse events (TEAEs) recorded through 28 days from start of consent to post‐treatment discontinuation.

### 5.4 Outcomes

The primary endpoint was PFS, defined as the duration from first intervention administration to disease progression or death from any cause. Secondary endpoints include ORR, disease control rate (DCR), duration of response (DOR), OS, and safety assessments. Overall response rate (ORR) was defined as the percentage of patients with at least one confirmed complete (CR) or partial response (PR) before progression. OS was defined as the time from treatment initiation to death from any cause. DCR was defined as the percentage achieving CR, PR, or stable disease (SD) as the best overall response. DOR was the period from initial response to documented progression or death without progression.

### 5.5 Statistical Analysis

Continuous variables were presented as mean ± standard deviation (SD) or median (range), whereas categorical variables were reported as frequencies and percentages. Kaplan–Meier (KM) analysis was utilized to estimate survival endpoints with corresponding 95% CI. Swimmer's plot and KM curves are plotted using Graph Pad Prism (version 10.6.1). Safety assessments concentrated on the incidence of TEAEs, with a particular emphasis on events of grade ≥ 3 severity. All statistical tests were two‐sided, with a significance threshold of *p* = 0.05, and analyses were conducted using SPSS v25.0 (IBM, Chicago, IL, USA) and R version 4.0.1.

## Author Contributions

Gen Lin and Qian Chu conceived and designed the study. Longfeng Zhang, Kang Miao, and Qian Miao collected and organized the clinical data. Dingzhi Huang and Zhe Liu conducted the statistical analysis. Long Huang, Yongfeng Yu, and Xiaobin Zheng contributed to the interpretation of the data. Yiquan Xu provided critical revisions to the manuscript. All authors reviewed and gave final approval for the version to be published and agreed to be accountable for all aspects of the work. All authors have read and approved the final manuscript.

## Funding

This work was supported by the National Natural Science Foundation of China (Grants No. 82072565 and No. 82372954, to G.L.), the Fujian Provincial Health Systemic Innovation Project (Grant No. 2020CXA010, to G.L.), the Scientific and Technological Innovation Joint Capital Projects of Fujian Province (Grant No. 2020Y9038, to G.L.), the Beijing Xisike Clinical Oncology Research Foundation (Grant No. Y‐2019AZZD‐0386, to G.L.), the Major Project for Young and Middle‐aged Scientists of Fujian Provincial Health Commission (Project No. 2023ZQNZD008, to G.L.), and the High‐Level Talent Training Project of the Fujian Cancer Hospital Committee (Project No. 2024YNG04, to G.L.).

## Ethics Statement

The study was performed in line with the principles of the Declaration of Helsinki and approved by the Ethics Review Committee of Fujian Medical University Cancer Hospital. Clinical experiment approval number: FJCH‐2021‐023. Informed consent was obtained from all patients.

## Conflicts of Interest

The authors declare no conflicts of interest.

## Supporting information



Supporting File 1: mco270855‐sup‐0001‐SuppMat.docx

## Data Availability

The data that supports the results of this study are available from the corresponding author upon reasonable request.
