# Supplementary material for: Safety and Efficacy of Aumolertinib as First‐Line Treatment in EGFR‐Mutant Lung Adenosquamous Carcinoma: A Multicenter, Single‐Arm, Prospective Phase II Study (ARISE Study)
Source: MedComm (2020). 2026 Jul 22;7(8):e70855. doi: 10.1002/mco2.70855 (PMC13390188; doi:10.1002/mco2.70855)
Supplement: Supplementary file 1 — Supporting File 1: mco270855‐sup‐0001‐SuppMat.docx [file MCO2-7-e70855-s001.docx]

**Safety and Efficacy of Aumolertinib as First-Line Treatment in EGFR-Mutant lung Adenosquamous Carcinoma: a Multicenter, Single-Arm, Prospective Phase II Study (ARISE Study)**

Longfeng Zhang^1^, Long Huang^2^, Dingzhi Huang^3^, Zhe Liu^4^, Yongfeng Yu^5^, Kang Miao^1^, Qian Miao^1^, Xiaobin Zheng^1^, Yiquan Xu^1^, Qian Chu^6^*, Gen Lin^4^*

**Table S1. Efficacy Subgroup Analysis of EGFR Mutation Subtypes**

| Efficacy Outcome | Ex19del  (n=7) | L858R  (n=5) |
| --- | --- | --- |
| Median PFS (95% CI) | 15.10 months (4.70-NA) | 4.27 months (3.33-NA) |
| 6-month PFS rate (%) | 71.40 | 40.00 |
| Median OS (95% CI) | NA months (11.30-NA) | 12.30 months (6.80-NA) |
| 12-month OS rate (%) | 71.40 | 60.00 |
| Confirmed ORR (%) | 71.40 (5/7) | 40.00 (2/5) |
| Confirmed DCR (%) | 85.70 (6/7) | 80.00 (4/5) |
| Median DoR (95% CI) | 10.8 months (4.2-NA) | 8.5 months (3.1-NA) |

Abbreviations: PFS, progression-free survival; OS, overall survival; ORR, objective response rate; DCR, disease control rate; DoR, duration of response; CI, confidence interval.

**Table S2. Efficacy Comparison of Key Studies on EGFR‑TKI Therapy for ASC**

| Study | Drug | Design | Sample Size | ORR (%) | Median PFS (months) | Median OS (months) |
| --- | --- | --- | --- | --- | --- | --- |
| ARISE Study (current) | Aumolertinib | Prospective, single arm | 12 | 58.3 | 11.1 | 16.7 |
| Lin et al.^1^ | First/second-generation TKIs | Retrospective, multicenter | 129 | 56.6 | 10.1 |  |
| Xu et al.^2^ | EGFR-TKIs | Retrospective | 15 | 60.0 | 8.08 |  |
| Xia et al.^3^ | EGFR-TKIs | Retrospective | 44 | 54.5 | 8.8 | 19.43 |
| Hu et al.^4^ | First-generation EGFR-TKIs  Third-generation TKI | Retrospective | 36  8 | 37.9  37.5 | 9.3  10.2 | 38.3 |

Abbreviations: ASC, Lung adenosquamous carcinoma; TKI, tyrosine kinase inhibitor; ORR, objective response rate; PFS, progression-free survival; OS, overall survival.

**References:**

1. Lin G, Li C, Li PS, et al. Genomic origin and EGFR-TKI treatments of pulmonary adenosquamous carcinoma. *Annals of Oncology*. 2020;31(4):4.

2. Xu J, Zhang Y, Jin B, et al. Efficacy of EGFR tyrosine kinase inhibitors for non-adenocarcinoma lung cancer patients harboring EGFR-sensitizing mutations in China. *J Cancer Res Clin Oncol*. 2016;142(6):1325-1330.

3. Xia X, Du W, Zhang Y, Li Y, Yu M, Liu Y. Efficacy of epidermal growth factor receptor-tyrosine kinase inhibitor for lung adenosquamous cell carcinoma harboring EGFR mutation: a retrospective study and pooled analysis. *Front Oncol*. 2024;14.

4. Hu M, Zhang B, Xu J, et al. Clinical Outcomes of Different Generations of EGFR Tyrosine Kinase Inhibitors in Advanced Lung Adenosquamous Carcinoma. *Mol Diagn Ther*. 2019;23(6):773-779.

**Figure S1.** Distribution of treatment-related adverse events (TRAEs) by CTCAE grade (n=9). X-axis: TRAE types; Y-axis: number of patients. Light gray bars represent grade 1-2 TRAEs, dark gray bars represent grade 3-4 TRAEs. The most common grade 1-2 TRAEs were elevated AST (3 patients) and myalgia (3 patients); grade 3-4 TRAEs included neutropenia, hypokalemia, elevated GGT, and hyponatremia (1 patient each).
